# Ultra-low dose immunization and multi-component vaccination strategies enhance protection against malaria in mice

**DOI:** 10.1038/s41598-021-90290-8

**Published:** 2021-05-24

**Authors:** Katharine A. Collins, Florian Brod, Rebecca Snaith, Marta Ulaszewska, Rhea J. Longley, Ahmed M. Salman, Sarah C. Gilbert, Alexandra J. Spencer, David Franco, W. Ripley Ballou, Adrian V. S. Hill

**Affiliations:** 1 Jenner Institute, Nuffield Department of Medicine, University of Oxford, Oxford, UK; 2 GSK Vaccines, Rixensart, Belgium; 3 Present Address: Radboud Institute for Health Science, Radboud University Medical Center, Nijmegen, The Netherlands

**Keywords:** Malaria, Immunology, Infectious diseases, Vaccines

## Abstract

An effective vaccine would be a valuable tool for malaria control and elimination; however, the leading malaria vaccine in development, RTS,S/AS01, provided only partial protection in a Phase 3 trial. R21 is a next-generation RTS,S-like vaccine. We have previously shown in mice that R21 administered in Matrix-M is highly immunogenic, able to elicit complete protection against sporozoite challenge, and can be successfully administered with TRAP based viral-vectors resulting in enhanced protection. In this study, we developed a novel, GMP-compatible purification process for R21, and evaluated the immunogenicity and protective efficacy of ultra-low doses of both R21 and RTS,S when formulated in AS01. We demonstrated that both vaccines are highly immunogenic and also elicit comparable high levels of protection against transgenic parasites in BALB/c mice. By lowering the vaccine dose there was a trend for increased immunogenicity and sterile protection, with the highest dose vaccine groups achieving the lowest efficacy (50% sterile protection). We also evaluated the ability to combine RTS,S/AS01 with TRAP based viral-vectors and observed concurrent induction of immune responses to both antigens with minimal interference when mixing the vaccines prior to administration. These studies suggest that R21 or RTS,S could be combined with viral-vectors for a multi-component vaccination approach and indicate that low dose vaccination should be fully explored in humans to maximize potential efficacy.

## Introduction

The malaria parasite has a highly complex lifecycle and vaccination strategies are being developed to target the parasite during each distinct stage^1^. The *Plasmodium* lifecycle begins in the human host with the pre-erythrocytic stage, where sporozoites invade hepatocytes and replicate to produce many thousands of daughter merozoites. Eliminating parasites at this stage is an attractive approach for malaria vaccination as it has the potential to prevent the progression to blood-stage infection and clinical disease, and also prevent onward transmission. A range of subunit and whole sporozoite vaccination approaches are being explored to target this stage, with varying levels of success achieved to date^1^. The most advanced malaria vaccine in clinical development is RTS,S, a particle vaccine targeting the circumsporozoite protein (CSP) which is highly abundant on the sporozoite surface^2^. RTS,S is most immunogenic when administered in the highly potent adjuvant, AS01^3,4^; but when recently evaluated in a Phase 3 clinical trial it was only partially protective, with some evidence of concerning safety signals and a possible negative impact on all-cause mortality in females^5^. During the first 18 months of follow-up, 3 doses of RTS,S/AS01 induced protective efficacy against clinical malaria of 46% in 5–17 month old children, and 27% in 6–12 week old infants^6^. This protective efficacy declined during the 38–48 month follow up and at the end of the trial was 28.3% and 18.3% for the children and infants, respectively^7^. Thus, a vaccine able to elicit higher and more durable efficacy is still a major priority.

We previously developed R21^8^ as a next generation RTS,S-like vaccine with the aim of enhancing protective efficacy by generating a more immunogenic CSP-based particle vaccine. Both RTS,S and R21 particles are based on a CSP-Hepatitis B surface antigen (HBsAg) fusion protein, with HBsAg being the carrier matrix that enables particle formation and presentation of CSP epitopes on the particle surface. R21 differs from RTS,S in that it contains only the CSP-HBsAg fusion protein, whereas RTS,S has a four-fold molar excess of HBsAg compared to the fusion protein. R21 therefore likely displays more CSP on the surface (Figure 4A) consistent with potent induction of HBsAg antibodies by RTS,S but not R21^8^. This could potentially lead to improved induction of CSP-specific antibodies by R21. We previously demonstrated that R21 is highly immunogenic, and when formulated with the ISCOM-based adjuvant Matrix-M^9^, it induces sterile protection in BALB/c mice^8^.

In contrast to the blood-stage of infection, during the pre-erythrocytic stage there are very few parasites (sporozoites and infected hepatocytes) present, making this an attractive bottleneck during the malaria parasite lifecycle for targeting and eliminating parasites^10,11^. However, since immune evasion by only a single sporozoite could result in a blood-stage infection^12^, anti-sporozoite vaccines are often considered ‘leaky’. Even the highest levels of vaccine-induced immune responses to a single antigen may not be sufficient to confer complete sterile protection in humans. It is therefore possible that a multi-component vaccination strategy may be required to achieve high levels of protection^13,14^. For this reason we previously evaluated R21 in combination with another clinically advanced pre-erythrocytic stage vaccine regimen—ChAd63—MVA viral vectors encoding ME.TRAP—which aims to induce T cells that target infected hepatocytes^15–18^. It is hypothesized that if the R21-induced, CSP-specific antibodies can eliminate most of the sporozoites before they enter the liver cells, then this increases the chance that TRAP-specific T cells can clear the remaining parasite infected hepatocytes and prevent blood-stage infection^19,20^. We have shown that both R21 and ChAd63—MVA ME.TRAP are immunogenic in BALB/c mice when administered alone. When administered together the result is concurrent induction of high CSP-specific antibody titers and high frequencies of TRAP-specific T cells and also enhanced protective efficacy^8^.

Here we evaluated whether RTS,S/AS01 can also be combined successfully with TRAP-based viral-vectors in mice, and if this can result in enhanced protective efficacy, as observed with R21. We describe a novel, Good Manufacturing Practice (GMP)-compatible purification process for R21 that uses a 4 amino acid (E-P-E-A) C-terminal tag (C-tag) fused to the R21 fusion protein (Figure 4A) to enable purification by affinity chromatography on the CaptureSelect affinity matrix^21^. In addition, we assessed the immunogenicity of ultra-low doses of R21 and RTS,S administered in the same adjuvant (AS01) and evaluated the protective efficacy of these vaccines in BALB/c mice using transgenic *P. berghei* sporozoites expressing *P. falciparum* CSP.

## Results

### RTS,S can be combined with TRAP-based viral-vectors

We have previously shown in BALB/c mice that R21 can be successfully combined with TRAP based viral-vectors without reducing immunogenicity of either vaccine^8^. Here, in two separate experiments, we evaluated the ability to combine RTS,S with TRAP based viral-vectors with either a ‘staggered’ administration approach, ‘co-administration’ of the vaccines at different sites, or ‘mixing’ the vaccines together prior to administration (Tables 1, 2).

**Table 1:** Immunisation regimens for comparing individual vaccines to co-administration.

Regimen	Group	Day 0	Day 7	Day 14	Day 28	Day 42
Individual immunisation regimens	A-M			ChAd		MVA
	A-M-M			ChAd	MVA	MVA
	R-R-R(5 µg)			RTS,S	RTS,S	RTS,S
Staggered immunisation	R-A-R-R-M	RTS,S	ChAd	RTS,S	RTS,S	MVA
Co-administration	AR-R-MR			ChAd/RTS,S	RTS,S	MVA/RTS,S
	AR-MR-MR			ChAd/RTS,S	MVA/RTS,S	MVA/RTS,S

R = RTS,S/AS01, A = ChAd63 ME.TRAP, M = MVA ME.TRAP.

**Table 2:** Immunisation regimens for co-administration and mixture comparison.

Group	RTS,S dose (µg)	Day 0	Day 14	Day 28
R-R-R	1.6	RTS,S	RTS,S	RTS,S
AR-MR-MR co-ad		ChAd/RTS,S	MVA/RTS,S	MVA/RTS,S
AR-MR-MR mix		ChAd + RTS,S	MVA + RTS,S	MVA + RTS,S
R-R-R	5	RTS,S	RTS,S	RTS,S
AR-MR-MR co-ad		ChAd/RTS,S	MVA/RTS,S	MVA/RTS,S
AR-MR-MR mix		ChAd + RTS,S	MVA + RTS,S	MVA + RTS,S

R = RTS,S/AS01, A = ChAd63 ME.TRAP, M = MVA ME.TRAP, co-ad = co-administration of vaccines at separate sites, mix = mixing vaccines prior to administration.

**Figure 1:**
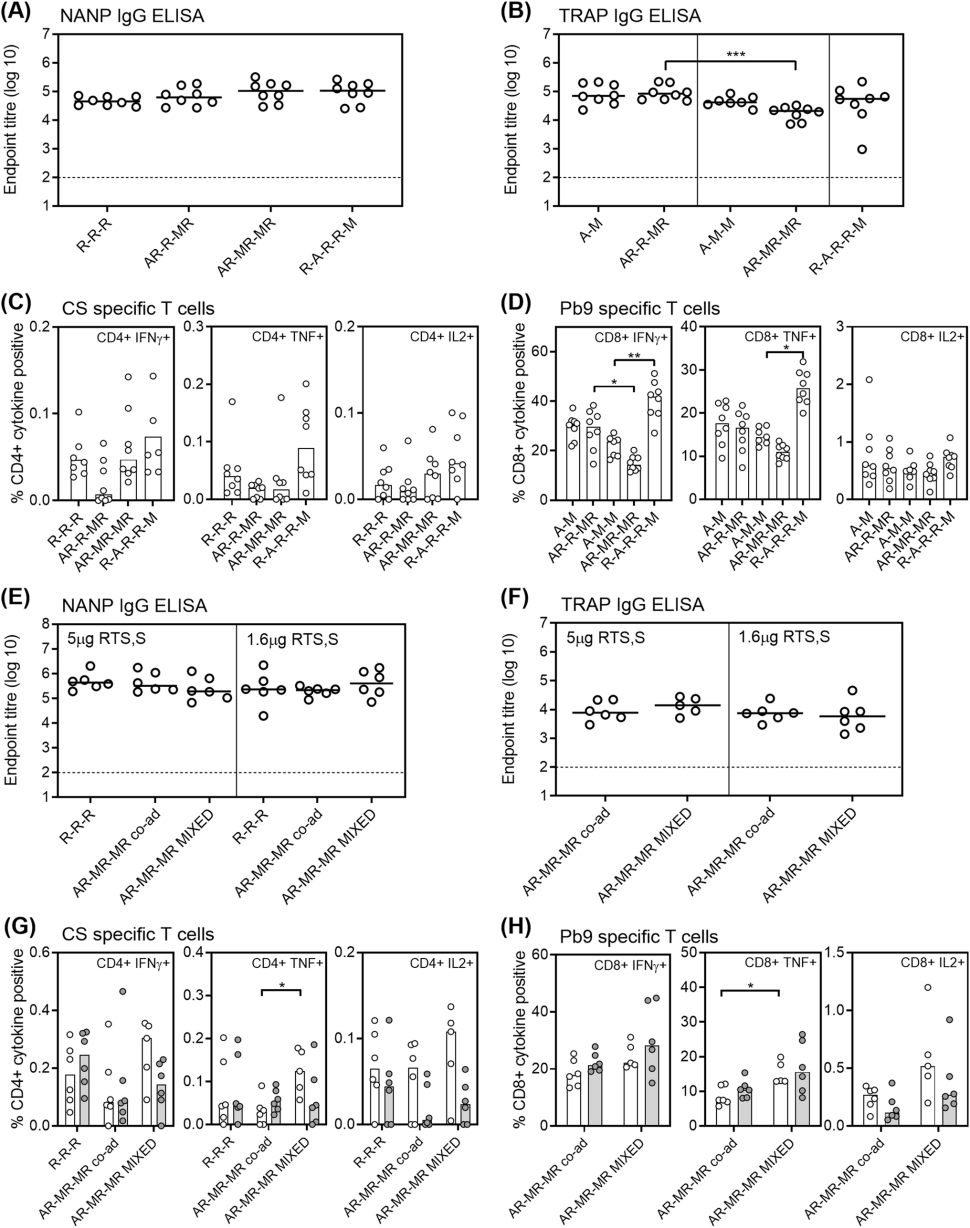
Minimal immunological interference when RTS,S/AS01 and ME.TRAP based viral vectors are co-administered at different sites or mixed together. BALB/c mice were immunized with RTS,S/AS01 and/or ChAd63 ME.TRAP—MVA ME.TRAP either alone, staggered or co-administered in different limbs, as indicated in Table 1. Two weeks after the final immunization (**A**) NANP-specific IgG responses and (**B**) TRAP-specific IgG responses were measured by ELISA, lines indicate the medians. T cell responses were measured in the blood ~ 2 weeks after the final immunization by ICS for (**C**) CSP-specific cytokine secreting CD4 + T cells or (**D**) Pb9-specific cytokine secreting CD8 + T cells, bars indicate the medians. BALB/c mice were immunized with RTS,S/AS01 and/or ChAd63 ME.TRAP—MVA ME.TRAP either alone, co-administered or mixed before immunization, as indicated in Table 2 using two doses of RTS,S/AS01 (5 µg or 1.6 µg) . Two weeks after the final immunization (**E**) NANP-specific IgG responses and (**F**) TRAP-specific IgG responses were measured by ELISA, lines indicate the medians. T cell responses were measured in the blood ~ 2 weeks after the final immunization by ICS for (**G**) CSP-specific cytokine secreting CD4 + T cells or (**H**) Pb9-specific cytokine secreting CD8 + T cells. Open bars and circles represent the 5 µg dose groups and grey bars and circles represent the 1.6 µg dose groups. Bars indicate the medians. Dotted lines indicate average background response. R = RTS,S/AS01, A = ChAd63 ME.TRAP, M = MVA ME.TRAP. All groups compared by Kruskal–Wallis with Dunn’s multiple comparison post-test comparing selected groups (*P < 0.05, **p < 0.01 and ***p < 0.0001).

In the first experiment, there was no decrease in RTS,S/AS01 immunogenicity when the vaccines were co-administered into different limbs (Figure 1A,C). Similarly, for TRAP-specific immunogenicity, there was no reduction in the TRAP-specific antibodies with co-administration, but there was a small non-significant reduction in ME.TRAP-specific CD8+ T cells (Figure 1B,D). Three immunizations with viral vectors (A-M-M) was less immunogenic than the normal prime boost regimen (A-M) when administered alone or in combination with RTS,S/AS01, but this was not statistically significant. Staggered administration of the vaccines resulted in a small non-significant increase in CSP-specific CD4+ T cells and a significant increase in IFNγ+ and TNF+ ME.TRAP-specific CD8+ T cells. CD4+ ME.TRAP-specific T cells and CD8+ CSP-specific T cells were also measured, but responses were similar to background so data is not shown.

The second experiment compared vaccine ‘co-administration’ at separate sites to ‘mixing’ the vaccines prior to administration, using 2 different doses of RTS,S (1.6 µg and 5µg). Induction of TRAP-specific and CSP-specific antibodies was unaffected by mixing the vaccines together compared to co-administration, and there was no difference between doses (Figure 1E,F). There was a consistent trend for enhanced ME.TRAP-specific CD8+ T cell responses with mixture vaccination and this was significant for TNF+ CD8 T cells (Figure 1H). There was also a consistent trend for increased CSP-specific CD4+ T cell responses with mixture vaccination and this was most apparent in the 5µg dose groups and significant for TNF+ CD4+ T cells (Figure 1G). CD4+ ME.TRAP-specific T cell and CD8+ CSP-specific T cell responses were negligible so the data is not shown.

### Efficacy of RTS,S can be enhanced by combination with TRAP based viral vectors

There was minimal immunological interference when RTS,S/AS01 was combined with ChAd63—MVA ME.TRAP in BALB/c mice in the previous experiments (Figures 1). A small non-significant reduction in ME.TRAP- and CSP-specific T cell responses was observed with co-administration and a small increase in ME.TRAP- and CSP-specific T cells was seen with mixture vaccination. This was evaluated using the clinical vaccine constructs ChAd63 ME.TRAP and MVA ME.TRAP. However, the ME string of these vaccines contains Pb9, the strong T cell epitope in *P. berghei* CSP that is able to confer sterile protection on its own, as seen in previous studies with high levels of induced Pb9 specific CD8+ T cells^22–24^. Thus, efficacy measured using these vaccines could reflect Pb9-specific T cell-elicited protection. In order to eliminate this potential confounding factor and to assess the protective efficacy of TRAP-specific immune responses, we used viral vectors expressing *P. berghei* TRAP without the ME string. To determine if combining the vaccines together results in increased efficacy we used C57BL/6 mice, a more stringent challenge model. This was because both RTS,S/AS01 and TRAP based viral vectors can elicit high levels of sterile protection when administered alone in BALB/c mice, making it impossible to detect a statistically significant increase in efficacy using reasonable numbers in the combination vaccine regimens. RTS,S/AS01 or the TRAP based viral-vectors were administered either alone or in combination, as detailed in Table 3. Malaria-naïve mice were also challenged as a control; adjuvant or vector only immunized mice were not included in an effort to reduce unnecessary use of mice as previous experiments in our laboratory demonstrate that they do not elicit any non-specific protection in this model^8,25,26^.

**Table 3:** Immunisation regimens used to evaluate efficacy of RTS,S/AS01 with TRAP based viral vectors.

Group	Day 0	Day 14	Day 28	Day 42
R-R-R	RTS,S	RTS,S	RTS,S	Transgenic P. berghei challenge TgPb + PfCSP
A-M-M	ChAd	MVA	MVA	
AR-MR-MR co-ad	ChAd/RTS,S	MVA/RTS,S	MVA/RTS,S	
AMR-AMR-AMR co-ad	ChAd/MVA/RTS,S	ChAd/MVA/RTS,S	ChAd/MVA/RTS,S	
AR-MR-MR mix	ChAd + RTS,S	MVA + RTS,S	MVA + RTS,S	
Naive	ChAd/RTS,S	MVA/RTS,S	MVA/RTS,S	

RTS,S = RTS,S AS01 A = ChAd63 Pb.TRAP, MVA = MVA Pb.TRAP, co-ad = co-administration of vaccines at separate sites, mix = mixing vaccines prior to administration.

**Figure 2:**
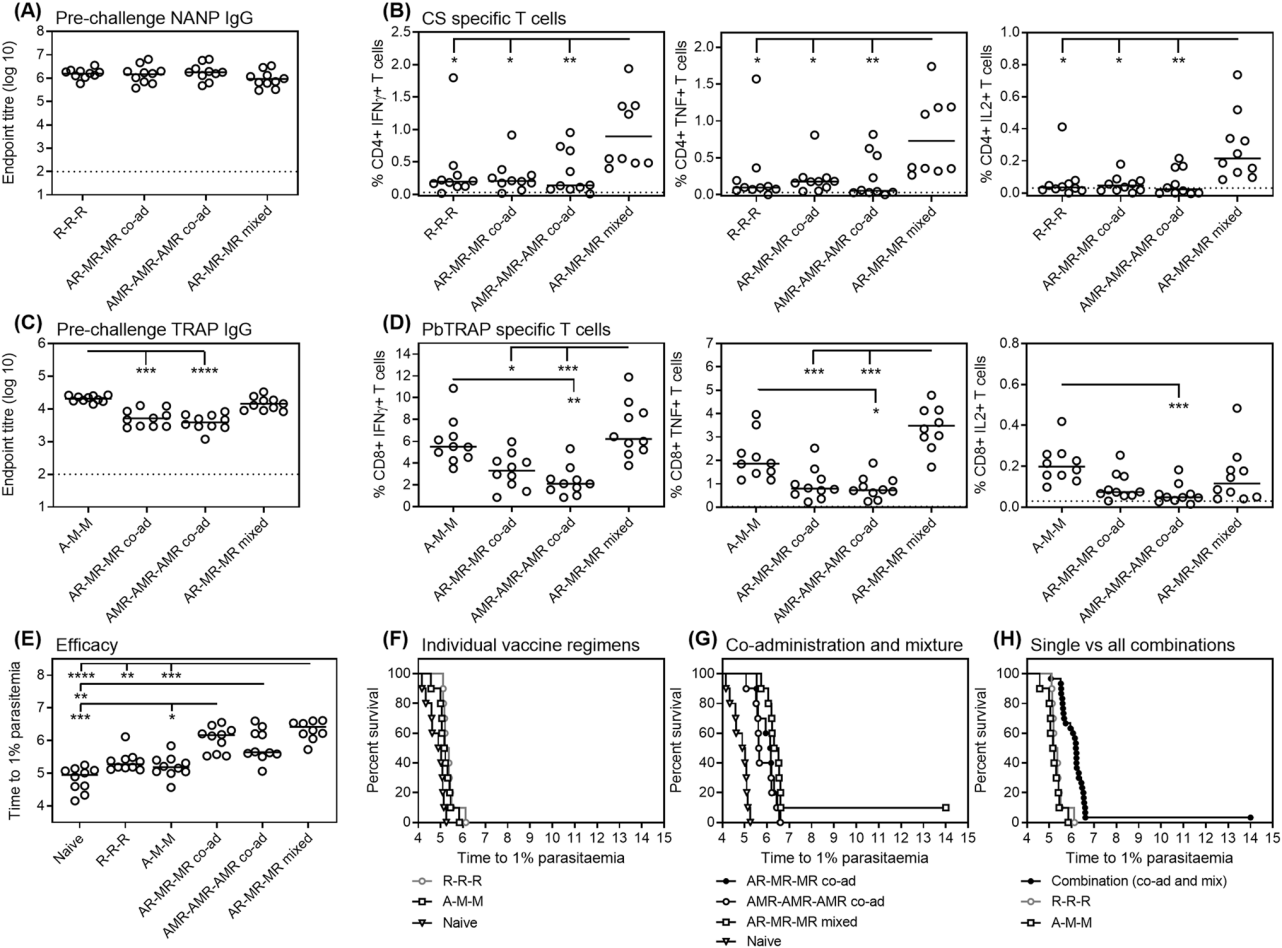
Immunogenicity and efficacy of RTS,S/AS01 and PbTRAP based viral-vector combinations. C57BL/6 mice were immunized with RTS,S/AS01 and/or ChAd63 PbTRAP—MVA PbTRAP either alone, co-administered or mixed as indicated in Table 3. Two weeks after the final immunization (**A**) NANP-specific IgG responses and (**B**) TRAP-specific IgG responses were measured by ELISA. Lines indicate the mean and groups compared by One-way ANOVA with Dunnett’s multiple comparison post-test comparing all pairs of groups. T cell responses were measured in the blood ~ 2 weeks after the final immunization by ICS for (**C**) CSP-specific cytokine secreting CD4 + T cells or (**D**) TRAP-specific cytokine secreting CD8 + T cells. Lines indicate the median. Dotted lines indicate average background response. Groups compared by Kruskal–Wallis test with Dunn’s multiple comparison post-test comparing all pairs of groups. Two weeks after the final immunization all immunized mice and 10 naïve mice were challenged with 1000 transgenic *P. berghei* parasites expressing *P. falciparum* CSP. Blood-stage parasitemia was monitored from day 5 after challenge by thin-film blood smear, and (**E**) time to 1% parasitemia was calculated using linear regression. Lines indicate the median and groups compared by Kruskal–Wallis test with Dunn’s multiple comparison post-test comparing all pairs of groups. The results are presented in Kaplan–Meier survival graphs and survival curves compared by Log-rank (Mantel–Cox) Test for (**F**) individual vaccinations, (**G**) combination and mixture vaccination, and (**H**) comparison of individual vaccination to all combinations. R = RTS,S/AS01, A = ChAd63 ME.TRAP, M = MVA ME.TRAP. *P < 0.05, **p < 0.01 and ***p < 0.0001.

In this experiment, adding TRAP based viral-vectors to RTS,S/AS01 did not interfere with the induction of NANP-specific IgG (Figure 2A), but did result in lower titers of PbTRAP-specific IgG. However, this reduction was only seen in the group where RTS,S and viral-vectors are co-administered at separate sites and not where they are mixed and administered in a single injection (Figure 2C). T cell responses in blood were measured by ICS with either CSP or PbTRAP peptides ~2 weeks after the final immunization. Mixing RTS,S/AS01 with viral vectors induced CD4+ CSP-specific T cell frequencies significantly greater than those induced by RTS,S/AS01 alone and also greater than those induced by vaccine co-administration (Figure 2B). There was no reduction in the PbTRAP-specific T cell frequencies with co-administration, and a significant increase observed with mixture vaccination (Figure 2D).

Following sporozoite challenge, there was a significant delay to development of 1% parasitemia in the groups receiving RTS,S/AS01 or the PbTRAP viral-vector regimen alone when compared to the naïve mice by Log-rank (Mantel–Cox) Test, *p* = 0.0004 and *p* = 0.0198, respectively (Table 4 and Figure 2F). All combination vaccine regimens also resulted in significant delay in development of 1% parasitemia when compared to the naïve mice (Table 4 and Figure 2E,G). The delay to 1% parasitemia was significantly greater for the combination regimens compared to RTS,S or the viral-vectors administered alone (*p* values range from 0.001 to < 0.0001 for all comparisons by Log-rank (Mantel–Cox) Test) (Figure 2E,G,H).

**Table 4:** Efficacy of RTS,S/AS01 and TRAP-based viral-vectors alone or combined.

Group	No. Protected/no. challenged	Time to 1% parasitemia (median)	Log-rank (Mantel–Cox) Test compared to naive
R-R-R	0/10 (0%)	5.28	*P* = 0.0004
A-M-M	0/10 (0%)	5.19	*P* = 0.0198
AR-MR-MR co-ad	0/10 (0%)	6.16	*P* < 0.0001
AMR-AMR-AMR co-ad	0/10 (0%)	5.64	*P* < 0.0001
AR-MR-MR mix	1/10 (10%)	6.42	*P* < 0.0001
Naive	0/10 (0%)	4.95	

RTS,S = RTS,S AS01 A = ChAd63 Pb.TRAP, MVA = MVA Pb.TRAP, co-ad = co-administration of vaccines at separate sites, mix = mixing vaccines prior to administration.

### R21 vaccine production can be optimized by addition of a C-tag allowing affinity purification

**Figure 3:**
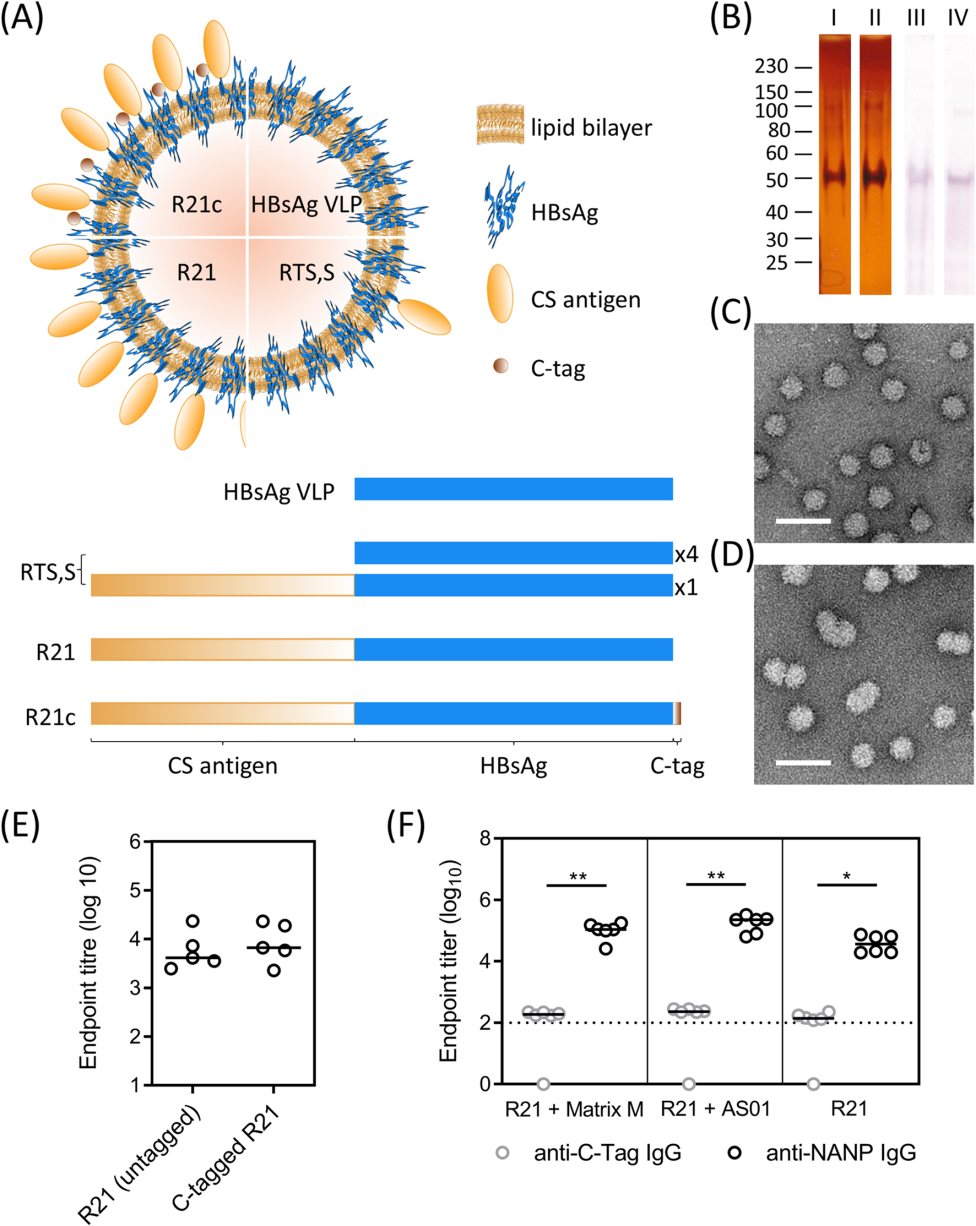
C-tagged R21 design and characterization. (**A**) The composition of fusion proteins making up the monomeric subunits of the particle vaccine products shown as bars and diagrammatic representation of the particle structure (R21c = C-tagged). (**B**) C-tagged R21 fusion proteins can be visualized by silver staining (lanes I and II) and anti-HBsAg western blot (lanes III and IV) after purification by C-Tag affinity chromatography (lanes I and III) followed by size exclusion chromatography (lanes II and IV). Transmission electron micrographs of (**C**) C-tagged R21 and (**D**) HBsAg particles after staining with 2% uranyl acetate. Scale bar = 50 µm. (**E**) NANP-specific IgG responses measured by ELISA in BALB/c mice 3 weeks after immunization with one dose of 0.5 µg C-tagged R21 (R21c) or untagged R21. (**F**) C-tag-specific and NANP-specific IgG responses measured by ELISA in BALB/c mice (n = 6 per group) immunized with 5 µg C-tagged R21 formulated either in Matrix-M or AS01 or without adjuvant (3 immunizations, 2 weeks apart). Individual data points shown with the group medians. Groups were compared by Kruskal–Wallis test with Dunn’s multiple comparison post-test. *P < 0.05, **p < 0.01 and ***p < 0.0001 Dotted line shows assay detection limit.

We have previously generated R21 particles by expressing the R21 fusion proteins in *Pichia pastoris*, lysing the yeast cells, and purifying the particles using their size and buoyant density^8^. Here, we evaluated a novel purification strategy with the aim of developing a GMP-compliant process using a C-terminal 4 amino acid tag (EPEA, termed “C-tag”) on the R21 monomer (Figure 3A). The C-tagged R21 particles were purified from the lysed yeast cell debris by affinity chromatography followed by a size exclusion chromatography polishing step isolating only the particles and removing monomeric fusion proteins. This process resulted in a highly pure R21 vaccine product, as demonstrated by silver staining and western blot analysis under reducing conditions (Figure 3B). A 50 kDa band was detected in the western blot, corresponding to the molecular weight of the R21 monomer, and in the silver stained gel an additional band was also detected at 100 kDa corresponding to the molecular weight of R21 fusion protein dimers that had not been completely reduced. Transmission electron micrographs of the vaccine product (stained with 2% uranyl acetate) show the C-tagged R21 particles were ~22 nm in size, comparable to the non-tagged R21 particles^8^ and HBsAg particles^27^. This demonstrates that the C-tag does not interfere with particle formation (Figure 3C,D). The purification process was also highly efficient, and yielded up to 14 mg of vaccine product per liter of yeast culture, ~10 fold more than non-tagged R21, thus superior to the initial purification strategy.

C-tagged R21 elicited comparable levels of CSP-specific antibodies compared to non-tagged R21 when both vaccines were formulated in Matrix-M and administered to BALB/c mice at the same dose (Figure 3E). Antibodies to the C-tag generated after vaccination were negligible compared to those induced to NANP (Figure 3F). All subsequent experiments in this study utilized C-tagged R21, which is referred to as R21 hereafter due to the characteristics being equivalent to the non-tagged R21.

### Low dose vaccination with R21 and RTS,S elicits high levels of immunogenicity and protective efficacy

**Figure 4:**
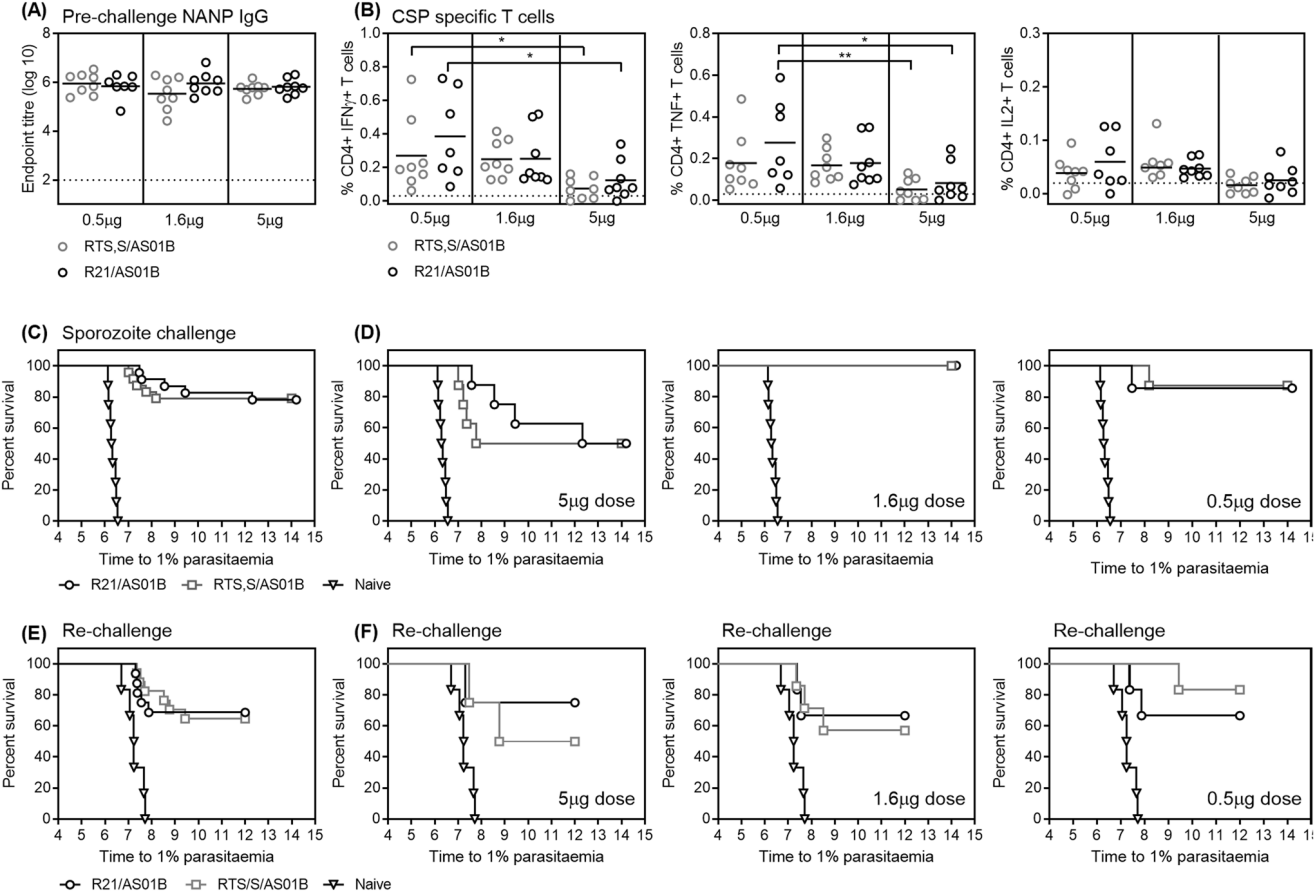
Immunogenicity and protective efficacy of low dose R21/AS01 and RTS,S/AS01. BALB/c mice (n = 8 per group, except 0.5 µg R21/AS01: n = 7) received 2 immunizations, 2 weeks apart of either R21/AS01 or RTS,S/AS01 at range of doses (0.5 µg, 1.6 µg or 5 µg). Two weeks after the final immunization (**A**) NANP-specific IgG was measured by ELISA, and (**B**) CSP-specific, cytokine secreting T cells (IFNγ + , TNF + or IL2 +) were measured by ICS and background responses subtracted. Lines indicate the mean and groups compared by One-way ANOVA with Turkey’s multiple comparison post-test comparing all pairs of groups. Dotted lines indicate average background response. Two weeks after the final immunization all immunized mice and 8 naïve mice were challenged with 1000 transgenic *P. berghei* parasites expressing *P. falciparum* CSP. Blood-stage parasitemia was monitored from day 5 after challenge by thin-film blood smear, and time to 1% parasitemia calculated using linear regression. Results are presented in Kaplan–Meier survival graphs and survival curves compared by Log-rank (Mantel–Cox) Test. (**C**) Comparison of protective efficacy between all mice vaccinated with either RTS,S/AS01 (n = 24) or R21/AS01 (n = 23). (**D**) Protective efficacy compared between RTS,S/AS01 and R21/AS01 at the different doses. Six months after the first challenge the surviving mice were re-challenged as before along with 6 naïve mice. (5 µg RTS,S/AS01: n = 4, 1.6 µg RTS,S/AS01: n = 7, 0.5 µg RTS,S/AS01: n = 6, 5 µg R21/AS01: n = 4, 1.6 µg R21/AS01: n = 6, 0.5 µg R21/AS01: n = 6). (**E**) Comparison of protective efficacy after re-challenge between all mice vaccinated with either RTS,S/AS01 (n = 17) or R21/AS01 (n = 16). (**F**) Protective efficacy after re-challenge compared between RTS,S/AS01 and R21/AS01 at the different doses. (*P < 0.05, **p < 0.01 and ***p < 0.0001).

To evaluate immunogenicity of low dose R21 and RTS,S vaccination, BALB/c mice received different concentrations of either R21 or RTS,S (5 µg, 1.6 µg or 0.5 µg) formulated with AS01 (5 µg MPL/5 µg QS-21 per injected dose). Three doses were given 3 weeks apart, and 2 weeks after the final vaccination all mice were challenged with 1000 transgenic *P. berghei* sporozoites expressing *P. falciparum* CSP (TgPb+PfCSP). Levels of CSP-specific antibodies and CSP-specific T cells were assessed ~2 weeks after the final vaccination, just prior to sporozoite challenge. Malaria-naïve mice were also challenged as a control; adjuvant immunized mice were not included as previous experiments demonstrate that they do not elicit any non-specific protection in this model^8,26^. No difference was detected in the magnitude of the NANP-specific IgG titers between RTS,S and R21 immunized mice, and there was no increase in NANP-specific IgG with increasing dose (Figure 4A). To assess differences in antibody quality, both avidity and IgG isotype were measured. Avidity was assessed using a NaSCN dissociation ELISA, and the ratio of IgG isotypes (IgG2a and IgG1) were measured as markers of Th1 and Th2 type responses. No differences in avidity or IgG isotype were detected between vaccines or doses (Figure S1A,B). CSP-specific T cells were measured in whole blood samples by ICS to a pool of overlapping CSP peptides. There was a trend to increased frequency of CD4+ CSP-specific T cells secreting IFNγ, IL2 or TNF with the lower dose of vaccine for both R21 and RTS,S and no difference between RTS,S and R21 immunized mice (Figure 4B). CD8+ CSP-specific T cell responses were similar to background, thus the data are not shown.

Following sporozoite challenge, both vaccines resulted in a significant delay to 1% parasitemia compared to the naïve mice (Figure 4C) and more than 50% of mice in each group were sterilely protected at each dose (Figure 4D). The protective efficacy of RTS,S/AS01 and R21/AS01 was equivalent when comparing the same vaccine dose groups, and in the 1.6µg dose groups 100% of the mice were sterilely protected. The highest dose of vaccine (5 µg) was significantly less protective than the lower doses, for both R21/AS01 and RTS,S/AS01, *p* = 0.03 (survival curves compared by Log-rank (Mantel–Cox) Test). Durability of this protective efficacy was evaluated by challenging the surviving mice a second time, six months after the first challenge. Very high levels of sterile protection were observed for both vaccines (Figure 4E) with no effect of dose (Figure 4F). Protection was comparable for the RTS,S and R21 immunized mice, and when data from all dose groups were pooled, sterile protection was 69% (5/16) for R21 and 65% (6/17) for RTS,S.

### R21 and RTS,S are immunogenic and protective at ultra-low vaccine doses

**Figure 5:**
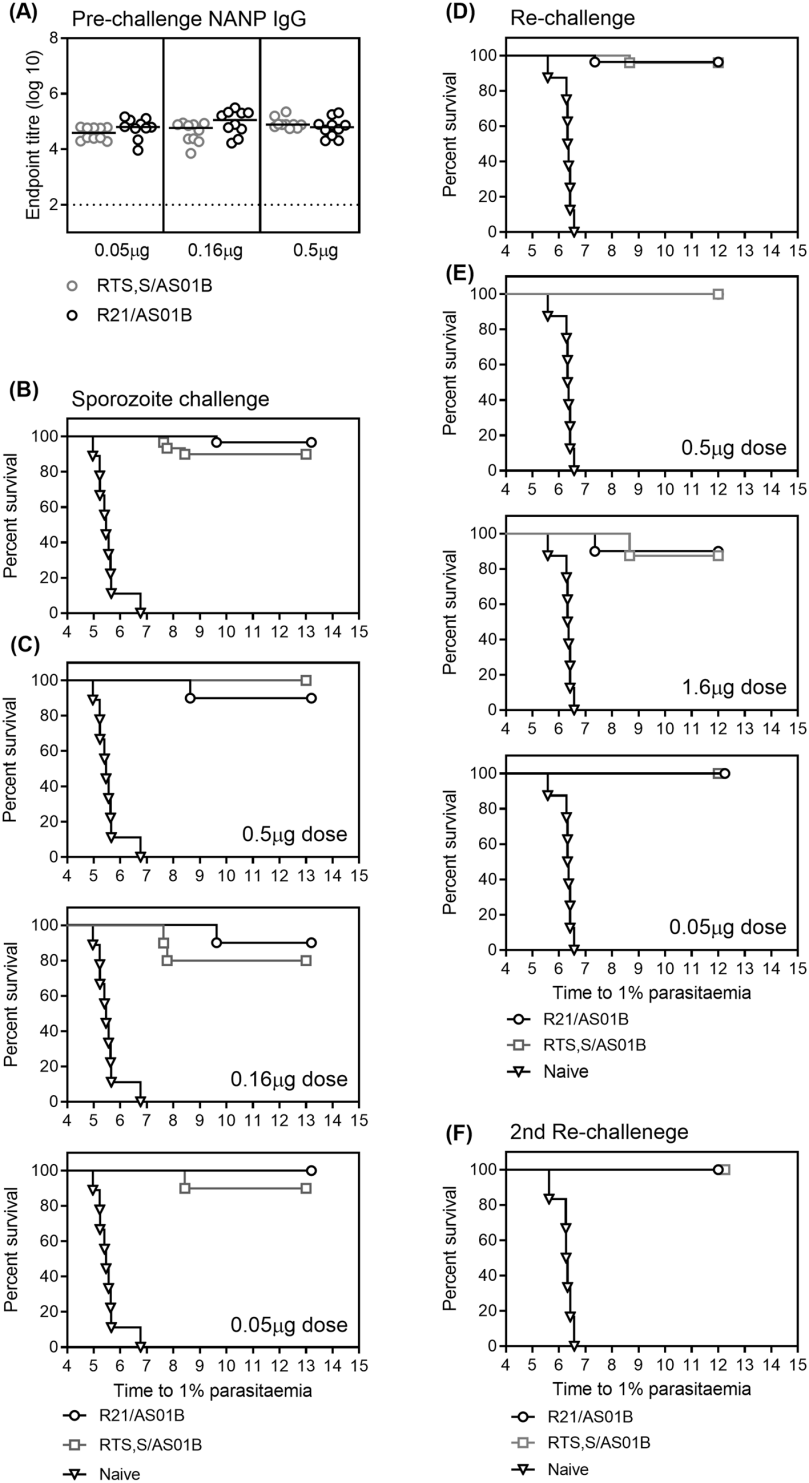
Immunogenicity and protective efficacy of ultra-low dose R21/AS01 and RTS,S/AS01. BALB/c mice (n = 10 per group) received 3 immunizations, 2 weeks apart of either R21/AS01 or RTS,S/AS01 at range of doses (0.05 µg, 0.16 µg or 0.5 µg). (**A**) Two weeks after the final immunization NANP-specific IgG was measured by ELISA. Lines indicate the medians and groups compared by Kruskal-Wallis test with Dunn’s multiple comparison post-test comparing all pairs of groups. At this time all immunized mice and 9 naïve mice were challenged with 1000 transgenic *P. berghei* parasites expressing *P. falciparum* CSP. Blood-stage parasitemia was monitored from day 5 after challenge by thin-film blood smear, and time to 1% parasitemia calculated using linear regression. The results are presented in Kaplan–Meier survival graphs and survival curves compared by Log-rank (Mantel–Cox) Test. (**B**) Comparison of protective efficacy between all mice vaccinated with either RTS,S/AS01 (n = 30) or R21/AS01 (n = 30). (**C**) Protective efficacy compared between RTS,S/AS01 and R21/AS01 at the different doses. Four months after the first challenge the surviving mice were re-challenged as before along with 8 naïve mice. (0.5 µg RTS,S/AS01: n = 10, 0.16 µg RTS,S/AS01: n = 8, 0.05 µg RTS,S/AS01: n = 7, 0.5 µg R21/AS01: n = 8, 0.16 µg R21/AS01: n = 10, 0.05 µg R21/AS01: n = 10). (**D**) Comparison of protective efficacy after re-challenge between all mice vaccinated with either RTS,S/AS01 (n = 25) or R21/AS01 (n = 28). (**E**) Protective efficacy after re-challenge compared between RTS,S/AS01 and R21/AS01 at the different doses. (**F**) Four months after the second challenge the surviving mice were re-challenged a second time as before along with 6 naïve mice RTS,S/AS01: n = 18 or R21/AS01: n = 21.

Due to the trend for increased protective efficacy with lower vaccine dose, ultra-low concentrations of R21 and RTS,S were evaluated as above (0.5 µg, 0.16 µg or 0.05 µg) with AS01 (5 µg MPL/5 µg QS-21 per injected dose) and the same immunization and challenge schedule. As seen before, there was no difference in the magnitude of the NANP-specific IgG titers detected between the R21/AS01 and RTS,S/AS01 groups and the antibody titers did not decrease with decreasing vaccine dose (Figure 5A). Following sporozoite challenge, at least 80% of the mice were sterilely protected in all groups and all doses of vaccine resulted in a significant delay to 1% parasitemia compared to naive mice. Protective efficacy was not reduced in the lower vaccine dose groups, and there was no difference in levels of efficacy elicited by R21/AS01 and RTS,S/AS01 (Figure 5B,C). Therefore, equivalent protective efficacy can be achieved in this model with a 100 fold reduction in vaccine dose with either R21/AS01 and RTS,S/AS01.

Durability of the protection elicited by ultra-low dose vaccination was assessed by re-challenging the protected mice a second (four months after first challenge) and third time (four months after second challenge). Sterile protection was very high, with 88% (51/53) of all mice protected in the second challenge (Figure 5D,E) and 100% (39/39) protected in the third challenge (Figure 5F). There was no difference in protective efficacy observed between vaccine dose groups or between the R21 and RTS,S groups. Antibody avidity, isotype and T cell responses were not measured in this experiment because no significant difference between vaccine dose groups was detected in the previous experiment.

### R21/AS01 and RTS,S/AS01 induced antibodies are durable

**Figure 6:**
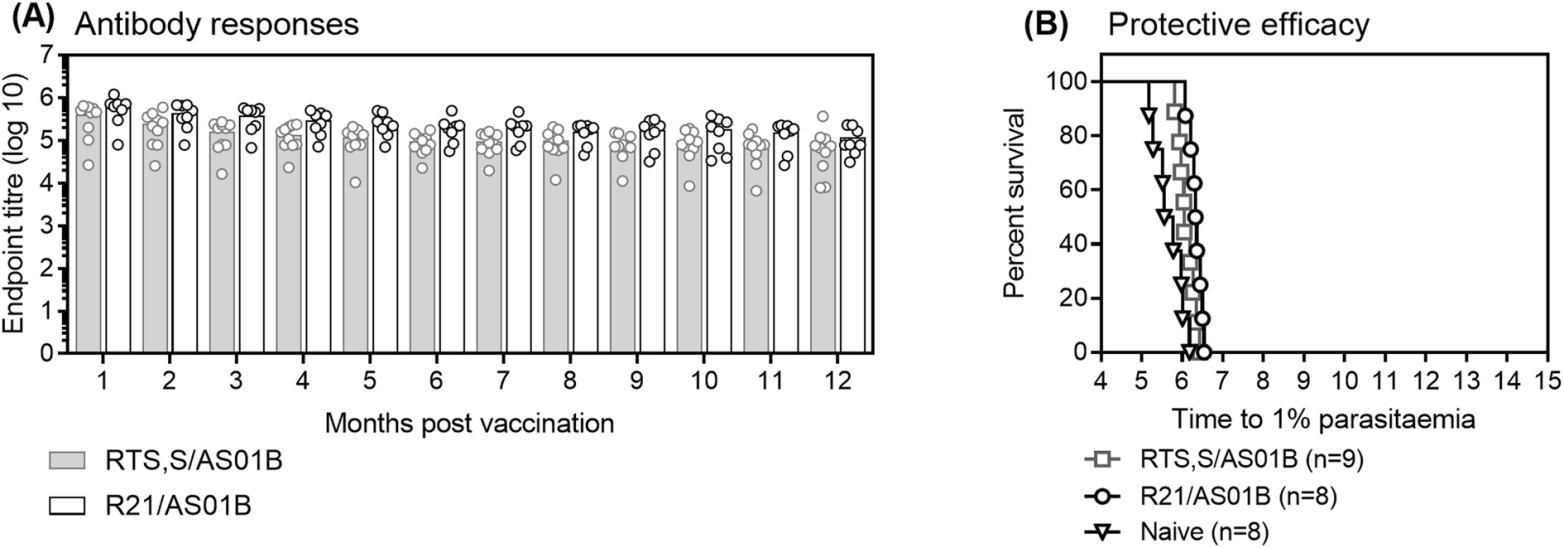
Durability of R21/AS01 and RTS,S/AS01 induced antibodies. BALB/c mice received 2 immunizations, 8 weeks apart of 1.6 µg of either R21/AS01 or RTS,S/AS01. (**A**) NANP-specific IgG was measured by ELISA every month after vaccination for one year. Bars represent mean with individual data points shown. One-way ANOVA with Dunnetts multiple comparison post-test comparing the titres at each time point to the titre at 1 month was used to evaluate reduction over time, and One-way ANOVA with Bonferroni multiple comparison post-test was used to compare R21 to RTS at each time point. (**B**) One year after the final immunization all surviving mice (R21: n = 8, RTS,S: n = 10) and 8 naïve mice were challenged with transgenic *P. berghei* parasites expressing *P. falciparum* CSP. Blood-stage parasitemia was monitored from day 5 after challenge by thin-film blood smear and time to 1% parasitemia calculated using linear regression. The results are presented in Kaplan–Meier survival graph and survival curves compared by Log-rank (Mantel–Cox) Test.

To evaluate the durability of RTS,S and R21 induced antibodies, BALB/c mice were immunized with two doses of either R21/AS01 or RTS,S/AS01 (1.6 µg) 8 weeks apart. This 2 dose immunization regimen was previously evaluated and conferred high levels of protection when R21 was formulated in Matrix-M or Abisco-100^8^. CSP-specific antibodies were measured to the NANP_6_C antigen by ELISA every month for a year following vaccination. NANP-specific IgG titers were stable in both groups. Twelve months after vaccination there was less than a log reduction in mean IgG titres (RTS,S: 5.61 to 4.96 and R21: 5.79 to 5.07) (Figure 6A). This reduction was significant for both vaccines (*p* = 0.01 for R21 and *p* = 0.004 for RTS,) and although the responses were slightly higher in the R21 compared to RTS,S vaccinated mice during the 12 months, there was no significant difference in the final NANP-specific IgG titers between the two groups. To determine the durability of protective efficacy, both groups of mice were challenged with 1000 TgPb+PfCSP sporozoites 12 months after vaccination. A significant delay in the development of 1% parasitemia was observed in both groups with no difference between the R21 and RTS,S immunized mice (Figure 6B). None of the mice were sterilely protected.

## Discussion

RTS,S/AS01 is the most advanced malaria vaccine candidate to date, and although completion of the Phase 3 clinical trial represented a major milestone in the development of an effective malaria vaccine, a more efficacious vaccine is still highly desirable. Here, we demonstrate that R21 when formulated in AS01 elicits comparable immunogenicity and protective efficacy to RTS,S/AS01 in a murine model. Moreover, we were able to enhance the efficacy of both vaccines with ultra low dose immunization, and both vaccines can be combined with TRAP based viral vectors for superior efficacy.

Both vaccines were shown to be highly immunogenic and able to elicit high levels of sterile protection (50–100%) at a range of vaccine doses. An increase in protective efficacy was observed with low dose vaccination (0.05 µg, 0.16 µg, 0.5 µg and 1.6 µg) compared to vaccination with a 5 µg dose. Improvements in efficacy by lowering vaccine dose have rarely been reported. A recent study evaluating PfSPZ irradiated sporozoite vaccine in Tanzania saw lower efficacy when vaccine dose was increased^28^. The mechanism for the increased efficacy remains elusive. The NANP-specific antibody titres were not greater in the groups with higher levels of sterile protection, suggesting efficacy is not mediated solely by the magnitude of the CSP-specific antibody titres. Initial analysis of antibody quality did not reveal any differences in either antibody avidity or the Th1-Th2 antibody isotype profile with the reduction in dose. Enhanced protection could be due to differences in the functional activity of the antibodies, and further evaluation using in vitro assays such as sporozoite invasion or liver-stage development assay may reveal important differences. The increased protection could also be due to the observed difference in CSP-specific CD4+ T cells since lower dose vaccination resulted in a trend for increased CSP-specific CD4+ T-cells (IFNg+ and TNF+). The specific contribution of CD4+ T cells to protection was not evaluated here, but this result is consistent with a previous R21 study where higher protective efficacy was achieved when R21 was administered in an adjuvant that induced higher levels of CSP-specific CD4+ T cells^8^. Other studies also report that CSP-specific T cells are associated with protective efficacy in murine models^29^, transgenic parasite models^30,31^, and in humans^4,32–34^; but exactly how they contribute to protection is still unclear.

Protection elicited by both vaccines was durable at all doses evaluated, as demonstrated by the re-challenge experiments. In these experiments protected mice were challenged a second or third time, 4 and 8 months after the initial challenge and in each group between 87.5 and 100% of the mice were sterilely protected. In agreement, only a modest reduction in NANP-specific antibody titres was observed in mice that were vaccinated and monitored for a year. However, when challenged for the first time one year after vaccination, none of these mice were sterilely protected. This suggests that the exposure to low doses of sporozoites during challenge experiments may have boosted or modified the vaccine induced immune responses resulting in the more durable efficacy. Whilst previous studies have shown that inoculation with large numbers of live sporozoites under drug cover can protect mice^35,36^, it is unlikely that the protection seen here is due solely to the sporozoite challenge, since a study in BALB/c mice required 20,000 sporozoites to achieve >90% sterile protection, and immunisation with a single dose of 4,000 sporozoites did not protect any mice^37^.

Combining RTS,S or R21, whose protective efficacy is mediated by targeting the sporozoites, with viral vectors that confer protection via T-cells targeting infected hepatocytes, might be a strategy to increase or maintain overall efficacy. We had previously shown that R21 can be combined with TRAP based viral vector vaccines with no reduction in immunogenicity and an enhancement in protective efficacy^8^. Here, we show that RTS,S/AS01 can also be combined with TRAP based viral vectors with minimal interference in induction of immune responses. There was a small reduction in responses with ‘co-administration’, but this was not always significant, and the reduction was not seen with ‘mixture’ vaccination. Efficacy was assessed in C57BL/6 mice, a strain in which it is frequently more difficult to achieve high level protection. In this mouse strain efficacy was also significantly greater in all groups where RTS,S was combined with the TRAP based viral vectors, and again this was most evident with ‘mixture’ vaccination as opposed to ‘co-administration’. This suggests RTS,S—like R21—could form the basis for a multi-component malaria vaccine. Combining RTS,S/AS01 with TRAP-based viral vectors was recently evaluated in two Phase1/2a trials using co-administration or concomitant administration but efficacy was not improved when evaluated by controlled human malaria infection 4 weeks after vaccination^38,39^. Our results here support further evaluation of combination regimens specifically evaluating low-dose vaccination and potentially mixing the vaccines together prior to administration as opposed to co-administration.

The affinity purification process established here has since enabled the efficient manufacture of R21 under GMP conditions, facilitating the clinical evaluation of R21 in a number of recent Phase I and 2a clinical trials in Oxford and west Africa. Interestingly, emerging data from clinical trials with a delayed and lower third dose of RTS,S (10 µg rather than 50 µg)^40^ and ongoing clinical trials of very low doses of R21 (Venkatraman et al. submitted for publication) suggest that lower doses of these HBsAg-based VLP vaccines expressing the CSP repeat might also be more protective in humans. R21 in the Matrix M adjuvant has now completed a one year follow-up in a Phase 2b trial in Burkina Faso, showing promising 77% efficacy against clinical disease with a three vaccination low dose regimen^41^. A Phase 3 trial of this R21 vaccine has recently started so that the two CSP immunogens compared here are currently the only two malaria vaccines to have entered Phase 3 trials.

## Methods

### C-tagged R21 vaccine purification

The gene for expression of R21c was cloned from the R21 expression plasmid^8^ into the PichiaPink expression plasmid pPink-HC using a reverse primer containing the C-Tag. Linearised plasmid DNA was transformed into electrocompetent PichiPink strain 1 cells. Yeast was grown as described previously^8^. Protein expression was induced with 1% methanol once per day. Cells were harvested by centrifugation and lysed in the presence of benzonase and detergent using glass beads. C-tagged proteins were purified from the lysates over a C-Tag affinity column prepared with 5 mL CaptureSelect C-tag Affinity Matrix (Thermo Scientific) packed into a XK16 column (GE Healthcare Life Sciences) with 2 M MgCl_2_ elution buffer. VLPs were further purified by size exclusion chromatography over a HiLoad 16/600 Superdex 200 pg column (GE Heathcare Life Sciences) using TBS as the running buffer.

### Protein characterization by SDS polyacrylamide gel electrophoresis

Vaccine samples were prepared in Laemmli buffer (BioRad) and proteins separated by SDS-PAGE on pre-cast NuPAGE 4-12% Bis-Tris Midi Protein Gels (Invitrogen) using NuPAGE MES SDS Running Buffer (Invitrogen). Total protein was either visualized by silver staining (Pierce Silver Stain Kit, Thermo Scientific) or blotted on nitrocellulose membranes using the BioRad TransBlot Turbo Transfer System (BioRad). Fusion proteins were detected using mouse anti-HBsAg (BioRad, MCA4658) and goat anti-mouse IgG-Alkaline Phosphatase antibody (Sigma, A3562). Western blots were developed with SIGMAFAST BCIP/NBT (Sigma).

### Animals and vaccinations

Six to ten week old female inbred BALB/c (H-2^d^) (BALB/cOlaHsd) mice or C57BL/6 (H-2b) (C57BL/6JOlaHsd) mice (Envigo, UK) were used as indicated and housed under Specific Pathogen Free (SPF) conditions. This study was carried out in accordance with the recommendations of the UK Animals (Scientific Procedures) Act 1986 and ARRIVE guidelines. Protocols were approved by the University of Oxford Animal Care and Ethical Review Committee for use under the UK Home Office granted Project Licenses PPL 30/2414 or 30/2889.

All vaccines were formulated, kept at ~4 °C until administration and delivered intramuscularly (i.m). For R21 the appropriate vaccine dose was formulated in a total volume of 25 µL endotoxin free, low phosphate PBS, mixed with 25 µL GSK proprietary liposome-based Adjuvant System AS01 containing 5 µg MPL and 5 µg QS-21 per injected dose, and was incubated for 30 minutes at 4 °C. QS-21 (*Quillaja saponaria* Molina, fraction 21) was licensed by GSK from Antigenics LLC, a wholly owned subsidiary of Agenus Inc., a Delaware, USA corporation.

For RTS,S vaccines depending on the dose, between 0.05 µL and 5 µL of RTS,S 1 mg/mL stock (GSK, Rixensart, Belgium) was mixed with AS01 (5 µg MPL/5 µg QS-21 per injected dose) for a 50 µL total injection volume and incubated for 30 minutes at 4 °C. For viral vector immunizations all vaccines were formulated using endotoxin free, low phosphate PBS in a total volume of 50 µL. The ChAd63 and MVA ME.TRAP and the ChAd63 and MVA PbTRAP constructs were generated as previously described^18,25^. Vaccine dose was: 1 × 10^8^ ifu ChAd63 ME.TRAP or ChAd63 PbTRAP and 1 × 10^6^ pfu MVA ME.TRAP or MVA PbTRAP. For R21/RTS,S and viral vector combination experiment mixture vaccinations were formulated together in a total volume of 100 µL and given in the same syringe (i.m.) split between both hind limbs, and for co-administration the vaccines were formulated separately and administered to a separate limb.

### Whole IgG ELISA

CSP and TRAP total IgG ELISAs were performed as previously described^8^. In brief, Nunc-Immuno Maxisorp 96 well plates were coated with antigen (either 2 μg/mL NANP_6_C peptide for CSP ELISAs, 1 μg/mL of PfTRAP or PbTRAP protein) and blocked. Sera were diluted at a starting concentration of 1:100 for samples post-prime or 1:1000 for samples post-boost, then serially diluted and incubated for 2 h at room temperature. Goat anti-mouse whole IgG conjugated to alkaline phosphatase was added for 1 h at room temperature. Plates were developed by adding p-nitrophenylphosphate at 1mg/mL in diethanolamine buffer, and OD was read at 405 nm. Serum antibody endpoint titers were taken as the x-axis intercept of the dilution curve at an absorbance value three standard deviations greater than the OD405 of serum from naïve mice. A standard positive serum sample was included in each assay as an assay control and a naïve serum sample was negative for antigen-specific responses to all antigens. C-Tag ELISAs were performed as CSP and TRAP ELISA, except half the plate was coated with the α-synuclein C-terminal peptide (YEMPSEEGYQDYEPEA) that contains the C-Tag sequence instead of NANP_6_C peptide and the same samples were added to both halves of the plate.

### Avidity ELISA

Avidity of anti-NANP_6_C antibodies was determined by chaotropic salt displacement ELISA. Serum samples whose endpoint titers had been determined previously were diluted to the dilution at which the OD405 in the endpoint ELISA had been 1. 50 μl of diluted sera were added to two columns of a Nunc-Immuno Maxisorp 96 well plate (Thermo Scientific) coated with 2μg/mL NANP_6_C peptide in carbonate-bicarbonate coating buffer (Sigma Aldrich). Plates were incubated for 2 h at room temperature, followed by washing and addition of increasing concentrations of NaSCN/PBS down the plate (0, 1, 2, 3, 4, 5, 6, and 7M NaSCN). Plates were incubated for 15 minutes at room temperature, washed and goat anti-mouse whole IgG conjugated to alkaline phosphatase added for 1 h at room temperature. Plates were developed by adding p-nitrophenylphosphate (Sigma) at 1mg/mL in diethanolamine buffer (Sigma) and OD was read at 405 nm. Avidity was given as the IC_50_ of NaSCN (concentration of NaSCN at which the signal is exactly half the intensity of the signal when no NaSCN was added).

### Isotype ELISA

Isotypes of anti-NANP_6_C antibodies were quantified using a standardized isotype ELISA. The top 6 rows of a Nunc-Immuno Maxisorp 96 well plate (Thermo Scientific) was coated with 2 μg/mL NANP_6_C peptide in carbonate-bicarbonate coating buffer (Sigma Aldrich). The two bottom rows were coated with a 1:2 dilution series of isotype control IgG (IgG2a or IgG1) in duplicate starting at 2 μg/mL. Plates were blocked with 10% Casein Block (Thermo Scientific). Sera were tested at two dilutions, each in triplicates. For anti-IgG1-Biotin, sera were diluted 1:10,000 and 1:40,000, and for anti-IgG2a-Biotin, sera were diluted 1:5,000 and 1:10,000. Diluted sera were added to the top six rows, and PBS was added to the bottom two rows. Plates were incubated for 2 h at room temperature washed and appropriate secondary antibodies were added at a dilution of 1:5000 (anti-mouse-IgG1-Biotin was added to the plate were mouse IgG1 was used for standard curve and anti-mouse-IgG2a-Biotin was added to the plate were mouse IgG2a was used). Plates were incubated for 1 h at room temperature, washed and Extravidine-AP was added at a dilution of 1:5,000. After 30 minutes of incubation the plates were washed and developed by adding 1mg/mL p-nitrophenylphosphate (Sigma) in diethanolamine buffer (Sigma) and OD_405_ was read. Plates were read when the 4^th^ IgG1 standard (0.25 μg/mL) and the 2nd IgG2a standard (1 μg/mL), respectively had reached an OD_405_ of 1.5. ELISA units for each Isotype were extrapolated from the samples OD on the linear range of the standard curve.

### Peptides

Peptides were prepared as previously described^8^. In brief, crude 20-mer peptides overlapping by 10 amino acids spanning the length of the PbTRAP vaccine insert sequence, or 15-mer peptides overlapping by 11 amino acids spanning the *P. falciparum* CSP sequence present in R21, were pooled into a single PbTRAP pool and a single CSP pool for ICS and ex vivo ELISpot assays. Pb9 (SYIPSAEKI)—the BALB/c immunodominant H-2Kd CD8+ epitope present in the multiple epitope (ME) string of the viral vector insert—was used to assess immune responses to the ME.TRAP vaccine insert.

### Blood ex vivo ICS

ICS was performed as previously described^8^. Briefly, PBMCs isolated from whole blood collected in EDTA were resuspended in complete α-MEM media and incubated in 96 well U bottom plates for 6 h at 37 with either GolgiPlug and complete α-MEM (as an unstimulated cell control) or GolgiPlug and peptide (1 µg/mL for Pb9 peptide or 5 µg/mL for CSP and PbTRAP peptide pools). PBMCs were washed and stained for 30 minutes on ice with 50 µL of surface stain mixture containing 1/50 Fc Block (CD16/CD32), 1/200 CD4 e450 and 1/200 CD8 Per CP Cy5.5 in PBS 0.5% BSA, followed by fixing and permeabilisation. Cells were then stained for 30 minutes on ice with 50 μl of intracellular stain mixture containing 1/100 TNF FITC, 1/100 IL2 PE and 1/200 IFNγ APC in Perm/Wash. Cells were acquired on the LSRII flow cytometer (BD Biosciences) and data were analysed in FlowJo (Tree Star Inc.). Results reported as the percentage of parent population (CD4+ or CD8+) secreting cytokine (TNF, IL2 or IFNγ) after unstimulated response is subtracted from the stimulated sample. Background response is the mean of the unstimulated response for the experiment.

### Sporozoite production and sporozoite challenge

The transgenic parasites were generated as previously described using the ‘gene insertion/marker out’ technology^42^. *P. berghei* transgenic parasites contained an additional copy of the *P. falciparum* CSP gene inserted at the 230p locus under the control of the *P. berghei* UIS4 promoter (TgPb+PfCSP). Transgenic sporozoites were produced using female *Anopheles stephensi* mosquitoes as previously described^8^. For all experiments 1000 sporozoites were injected intravenously (i.v.) in a total volume of 100 µL into the lateral tail vein of each mouse. From day 5 post challenge mice were monitored for infection by thin-film blood smear (fixed in methanol and stained in 5% Giemsa for 1 h). Mice were sacrificed after three consecutive parasite positive blood films. The time taken to develop 1% parasitemia was calculated using linear regression analysis for the parasite positive mice and if no parasites were detected on day 14 after challenge the mice were considered sterilely protected.

### Statistical analysis

Statistical analysis was performed using Graphpad Prism version 7, methods were as previously described^8^. In brief, where appropriate the D’Agostino-Pearson normality test was used to determine if the data were normally distributed. When comparing two groups or more the Kruskal-Wallis test with Dunn’s multiple comparison test was used for non-parametric data. Two or more groups of parametric data were compared by One-way ANOVA with Bonferroni’s multiple comparison test (when comparing all pairs of groups) or with Dunnett’s multiple comparison test (when comparing all groups to one group). Challenge results are presented in the Kaplan-Meier survival graphs and survival curves were compared by Log-rank (Mantel–Cox) Test. Significance was indicated when value of *p* < 0.05 (*p < 0.05, **p < 0.01, ***p < 0.001).

## Supplementary information

Supplementary Information.

## Data Availability

The raw data supporting the conclusions of this manuscript will be made available by the authors, without undue reservation, to any qualified researcher.
