# Supplementary material for: Ultra-low dose immunization and multi-component vaccination strategies enhance protection against malaria in mice
Source: Sci Rep. 2021 May 24;11:10792. doi: 10.1038/s41598-021-90290-8 (PMC8144388; doi:10.1038/s41598-021-90290-8)
Supplement: Supplementary file 1 — Supplementary Information. [file 41598_2021_90290_MOESM1_ESM.docx]

Ultra-low dose immunization and multi-component vaccination strategies enhance protection against malaria in mice

Katharine A Collins^1*+^, Florian Brod^1+^, Rebecca Snaith^1^, Marta Ulaszewska^1^, Rhea J Longley^1^, Ahmed M. Salman^1^, Sarah C Gilbert^1^, Alexandra Spencer^1^, David Franco^2^, W. Ripley Ballou^2^, Adrian VS Hill^1^

^1^Jenner Institute, University of Oxford, Oxford, UK.

^2^ GSK Vaccines, Rixensart, Belgium

^+^These authors contributed equally to this work

***Corresponding author:** Katharine A Collins
Email: [Katharine.a.collins@gmail.com](mailto:Katharine.a.collins@gmail.com)

Current address: Radboud Institute for Health Science, Radboud University Medical Center, Nijmegen, The Netherlands.

Keywords: Malaria, Vaccine, R21, RTS,S, Circumsporozoite protein, TRAP, transgenic, VLP, CSP.

**Supplementary Figures**

**
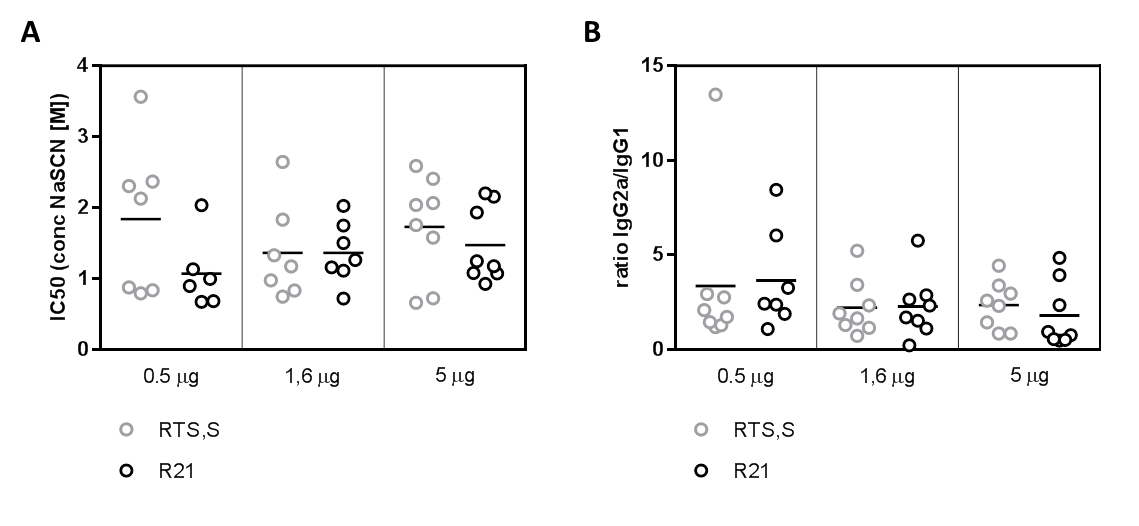
**

**Figure S1.** **Isotype and avidity ELISAs following low dose R21/AS01 and RTS,S/AS01**. BALB/c mice (n=8 per group, except 0.5 µg R21/AS01: n=7) received 2 immunizations, 2 weeks apart of either R21/AS01 or RTS,S/AS01 at range of doses (0.5 µg, 1.6 µg or 5 µg). Two weeks after the final immunization **(a)** anti-NANP_6_C antibody avidity was measured by chaotropic salt displacement ELISA and **(b)** antibody isotype was measured using a standardized isotype ELISA. Lines indicate the median and groups compared by Kruskal-Wallis test with Dunn’s multiple comparison post-test, no significant differences were found.


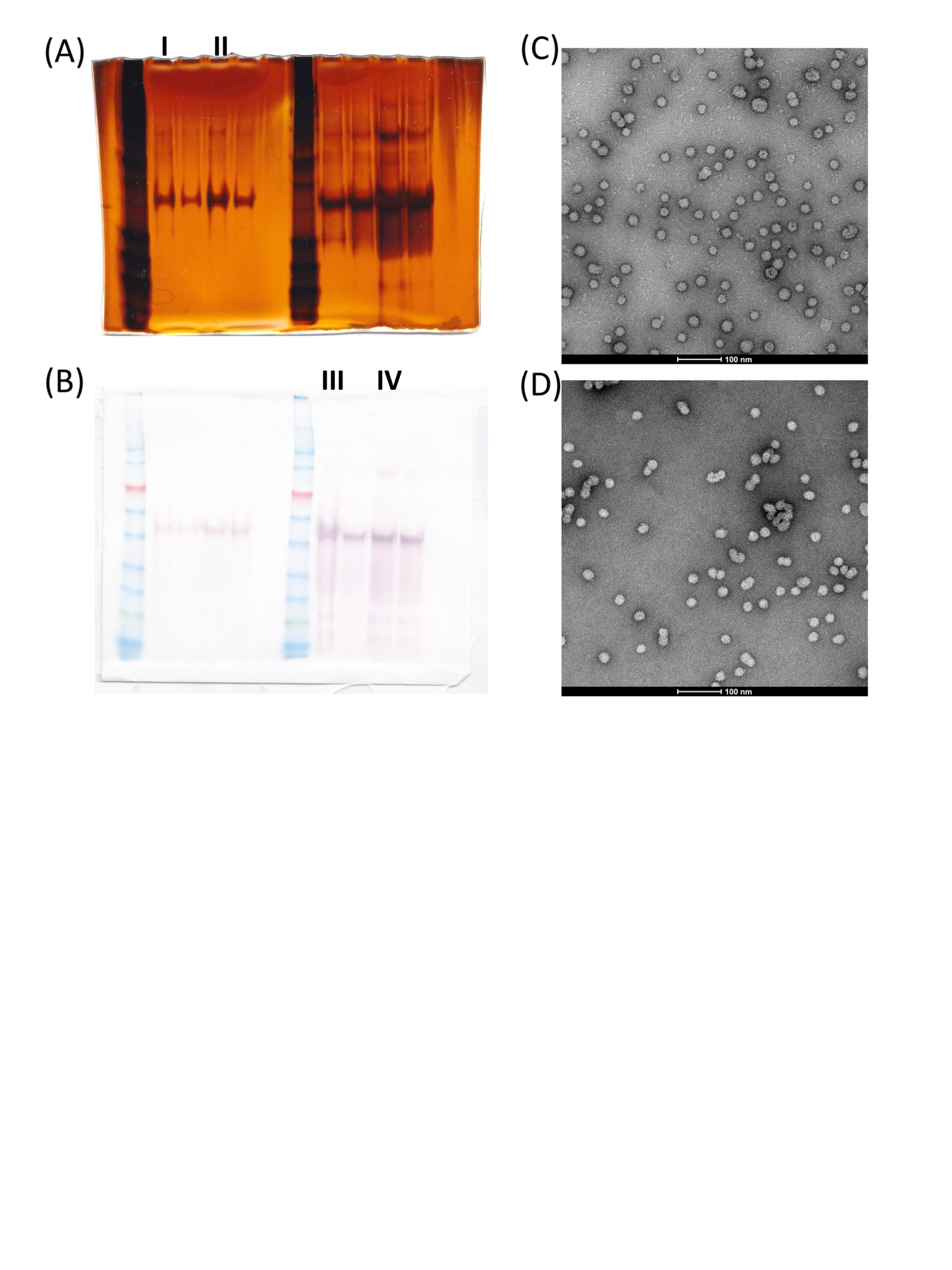


**Figure S2: Uncropped images for Figure 3.** **(a) and (b)** Silver stained gel and western blot of R21. Silver stained gel and western blot show the same samples, i.e two independent preparations of R21c purified by C-Tag affinity chromatography (lanes 1 and 2 after the marker) followed by size exclusion chromatography (lanes 3 and 4 after the marker). The left and right half of the gel and blot contain the same samples but the samples in the right are at twice the concentration than the left. Lanes shown in Figure 3 are indicated by the roman numericals. **(c) and (d)** Uncropped transmission electron micrographs of R21c **(c)** and HBsAg **(d)** VLPs shown in Figure 3.
